# Complex history of dog (*Canis familiaris*) origins and translocations in the Pacific revealed by ancient mitogenomes

**DOI:** 10.1038/s41598-018-27363-8

**Published:** 2018-06-14

**Authors:** K. Greig, A. Gosling, C. J. Collins, J. Boocock, K. McDonald, D. J. Addison, M. S. Allen, B. David, M. Gibbs, C. F. W. Higham, F. Liu, I. J. McNiven, S. O’Connor, C. H. Tsang, R. Walter, E. Matisoo-Smith

**Affiliations:** 1 Department of Anthropology and Archaeology, University of Otago, PO Box 56, Dunedin, 9054 New Zealand; 2 Department of Anatomy, University of Otago, PO Box 56, Dunedin, 9054 New Zealand; 3 Department of Human Genetics, David Geffen School of Medicine at UCLA, Los Angeles, 90024 United States of America; 4 Archaeology Department, American Samoa Power Authority, PO Box 2545, Pago Pago, AS 96799 American Samoa USA; 5 Anthropology, School of Social Sciences, University of Auckland, Private Bag 92019, Auckland, 1142 New Zealand; 6 Monash Indigenous Studies Centre, Monash University, 20 Chancellors Walk, Clayton, VIC, 3800 Australia; 7 School of Humanities, University of New England, Armidale, NSW 2351 Australia; 8 Institute of History and Philology, Academia Sinica, 128 Academia Rd, Taipei City 115, Taiwan; 9 Archaeology & Natural History, School of Culture History & Language, College of Asia & the Pacific, Australian National University, Acton, ACT 2601 Australia; 10 ARC Centre of Excellence for Australian Biodiversity & Heritage, Acton, ACT 2601 Australia

## Abstract

Archaeological evidence suggests that dogs were introduced to the islands of Oceania via Island Southeast Asia around 3,300 years ago, and reached the eastern islands of Polynesia by the fourteenth century AD. This dispersal is intimately tied to human expansion, but the involvement of dogs in Pacific migrations is not well understood. Our analyses of seven new complete ancient mitogenomes and five partial mtDNA sequences from archaeological dog specimens from Mainland and Island Southeast Asia and the Pacific suggests at least three dog dispersal events into the region, in addition to the introduction of dingoes to Australia. We see an early introduction of dogs to Island Southeast Asia, which does not appear to extend into the islands of Oceania. A shared haplogroup identified between Iron Age Taiwanese dogs, terminal-Lapita and post-Lapita dogs suggests that at least one dog lineage was introduced to Near Oceania by or as the result of interactions with Austronesian language speakers associated with the Lapita Cultural Complex. We did not find any evidence that these dogs were successfully transported beyond New Guinea. Finally, we identify a widespread dog clade found across the Pacific, including the islands of Polynesia, which likely suggests a post-Lapita dog introduction from southern Island Southeast Asia.

## Introduction

When people spread from west to east across the Pacific Ocean they did not travel alone. Often dogs (*Canis familiaris*), pigs (*Sus scrofa*), chickens (*Gallus gallus*) and rats (*Rattus exulans*) were part of these migrations. The presence of dog, pig, chicken and rat bones in archaeological sites attest to their inclusion in migratory voyages. However, moving domesticated animals across increasingly wider ocean gaps and onto often smaller, and more environmentally depauperate, islands of the Pacific, with no established agricultural economies, may have posed considerable challenges. The archaeological evidence for the introduction of domestic and commensal animals into the region is patchy and discontinuous^1,2^, which may signal some of the difficulties inherent in the establishment of viable animal populations, or indicate human choices made in relation to their transportation and management.

Explanations for the late Holocene human colonisation of Remote Oceania draw on archaeological and linguistic data to model the movement of Austronesian language speakers out of Taiwan and into the Pacific, via the Philippines. According to this model, this movement preceded the emergence of the Lapita Cultural Complex (LCC) in the Bismarck Archipelago which then spread across much of Near and Remote Oceania^3,4^. Archaeological evidence for the LCC is found from New Guinea to West Polynesia. Various scenarios have been suggested, incorporating archaeological, linguistic and biological evidence, to account for the dispersal processes and the extent to which interactions occurred between incoming Austronesian language speakers and existing human groups in Near Oceania (for a summary see^3^). Further to the east across the Pacific Ocean, dogs were part of the last major human migration that resulted in the initial colonisation of the islands of East Polynesia^4^.

Although East Asia is a critical region for dog domestication^5–7^, the subsequent translocation of dogs across Mainland Southeast Asia (MSEA) and Island Southeast Asia (ISEA) and into the islands of the Pacific is not well understood. Molecular genetic studies using mitochondrial DNA (mtDNA) that have investigated the origins and dispersals of dogs throughout the Pacific indicate that the dingo of Australia, New Guinea Singing Dog (NGSD) and ancient Polynesian dogs are all descended from East Asian dogs^8^. Despite their long association with people, dogs appear relatively late in the archaeological record of Oceania, and the timing of their arrival and dispersal trajectories appear to differ from that of people. The earliest archaeological dog remains are found in Australia and date to c. 3,500 years ago^9^, although this age is based on archaeological context and is not a direct date. Over time, the descendants of these dogs reverted to a completely independent state, and the population continued over successive generations without human involvement. These dogs, known as dingoes, are considered to be one of the few truly feral dog populations found anywhere in the world^10^. Nevertheless, some dingoes have been incorporated into Aboriginal communities as companions and hunting aides^11^. NGSD are genetically closely related to dingoes^8^. The history of these dogs is however little known and highly debated^12^. Modern lowland dogs in New Guinea today are potentially the result of admixture with introduced European breeds and may not be representative of true NGSD lineages; however, it is possible that NGSDs may still be present in isolated areas of the highlands.

Dog remains from archaeological sites provide an important source of information, not only about the dynamic relationship between dogs and people, but also to reconstructions of their genetic history. Using a 582 base pair (bp) portion of the control region of the mitochondrial genome (mitogenome) from modern dogs, Savolainen and colleagues demonstrated that all dingoes sampled belonged to a lineage known as the A29 haplotype^8^. Although this is one of a number of dog mtDNA lineages that reached ISEA, it was the only one successfully established in Australia. In addition, two short haplotypes (Arc1 and Arc2), were observed in archaeological dog remains from East Polynesia (sampled from Hawai’i, the southern Cook Islands and New Zealand), but not found in modern dingoes^8^. A later study by Oskarsson and colleagues investigated the origins and routes of the introductions of dingoes, NGSDs, and Polynesian dogs in further detail, using the same control region fragments as the previous study by Savolainen and colleagues^13^. The Arc1 haplotype was found to be indistinguishable from a number of widespread control region haplotypes found in modern dogs from China, MSEA and ISEA, while Arc2 had a much more restricted distribution, and appears to belong to the A75 lineage found only in modern Indonesian dogs. Oskarsson and colleagues compared the frequency and distribution of modern haplotypes in MSEA and ISEA, including Taiwan and the Philippines, with those from the dingo and ancient Polynesian samples. Neither the ancient Polynesian short haplotypes nor the A29 haplotype carried by dingoes were present in modern dogs sampled from Taiwan and the Philippines, suggesting that dogs were not introduced into the Pacific region from or via this northeastern route.

Second generation sequencing technology is now making it feasible to sequence the complete mitochondrial genome, rather than just a portion, and has also contributed to the viability of sequencing of mitogenomes from archaeological specimens^14^. As expected, studies of mitogenomes of modern domesticated species, such as cattle^15^ and sheep^16^, and from archaeological dog samples^17^ have shown that the control region sequences provide only a partial picture of genetic diversity. A recent study of dingoes using complete mitogenomes and nuclear markers has shown that there are at least two geographically subdivided genetic populations^18^, rather than the single population suggested by control region studies^8^.

**Figure 1:**
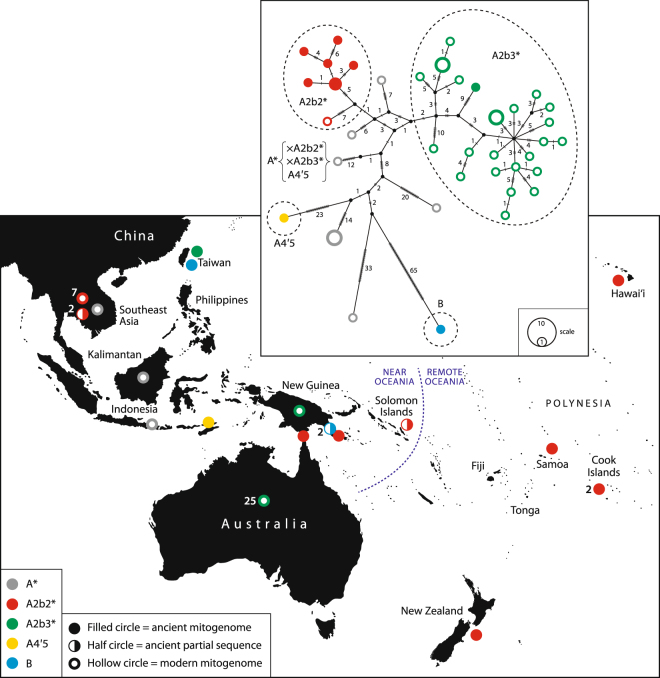
A map of Southeast Asia and the Pacific showing the source location of the specimens and associated haplogroups (assignment to haplogroup follows Duleba and colleagues^19^) and the median-joining network. The boundary between Near and Remote Oceania is also shown. Symbols identify the type of sequence: filled circle, ancient mitogenome; half circle, partial ancient sequence; hollow circle, modern mitogenome. Node colours represent the haplogroup, grey, A; red, A2b2, green, A2b3; yellow, A4’5; blue, B.

Here we present seven new complete and near-complete ancient mitogenomes and five partial ancient mitogenome sequences from archaeological dog specimens from Thailand, ISEA and the Pacific (Fig. 1, Supplementary Table S1), and mitogenomes from a sample of four modern dingoes from the Wellington Zoo in New Zealand (Supplementary Table S2). When combined with additional published mitogenomes from modern dogs from Southeast Asia, Australia and New Guinea (Supplementary Table S2), the aDNA data reveal a complex history of dog-human interactions in ISEA and the Pacific. Three temporally distinct dispersals are highlighted, and their possible origins and the sequence of introductions are revealed.

## Results

Phylogenetic analyses of complete mitogenomes of modern dogs from across the world have shown that over 65% of the dogs sampled fall within Haplogroup (Hg) A^19^. There is geographical structure within this large haplogroup, with the greatest mitochondrial diversity found in East Asia^5,19,20^. Duleba and colleagues^19^ further identified several sub-clades that are present almost exclusively in East Asian modern dogs. These sub-clades were estimated to have the oldest evolutionary ages (between 15,000 and 38,700 years ago) and are hypothesised to represent the initial founding gene pool. Here we follow Duleba and colleagues’ phylogeny and nomenclature for dog haplogroups, which has the greatest resolution for sub-clade A2, the lineage carried by the majority of dogs we sequenced in our study.

All the specimens from which we obtained mitogenome sequences belong to Hg A, with the exception of one specimen from Taiwan which belongs to Hg B and two partial sequences from specimens from the south coast of Papua New Guinea which also possess single nucleotide mutations (SNPs) consistent with Hg B (Fig. 1, and see Supplementary Table S1 for haplogroup assignments and archaeological site locations, Supplementary Table S3 for variable positions relating to haplogroup assignment, and Supplementary Table S4 for defining SNPs for Haplogroup B observed in the ancient Papua New Guinea sequences). Within Hg A, the majority of sequences from the ancient Pacific Island dogs and two partial sequences from Thai dogs, belong to Dubela and colleagues’^19^ Hg A2b2. These share most, but not all, of the defining mutations with a modern specimen from Fiji (KM061586 [11 ws] in^19^) located on one of the Hg A2b2 terminal branches. The Pacific Island sequences that share this A2b2 lineage span a wide area from New Guinea to East Polynesia (Fig. 1). They include mitogenomes obtained from dog remains from the archaeological site of Taurama, near Port Moresby, in Papua New Guinea^21^; the Goemu site on the island of Mabuyag in the Torres Strait which separates New Guinea and Australia^22^; a partial sequence from an archaeological site in Graciosa Bay, Santa Cruz in the Solomon Islands^23^; and complete mitogenomes from samples from sites on Tokelau in West Polynesia^24^ and Hawai’i^25^ in East Polynesia. One previously published ancient mitogenome from New Zealand^17^, and two from the southern Cook Islands^26^ also share this A2b2 lineage. The dingoes belong to Hg A2b3, and share only a few defining mutations with the specimen from China on one of the two A2b3 terminal branches (EU789681 in^19^). One ancient specimen, from Timor-Leste, belongs to Hg A4’5, and has some of the defining mutations for sub-clade A5.

The two nearly complete ancient mitogenomes from dogs from the Iron Age archaeological site of Shisanhang in Taiwan (MS10333, MS10334) are highly divergent compared to the other ancient sequences, representing the A and B haplogroups. One of the specimens (MS10333) carries the A2b3 haplotype, which is observable in modern dingoes but not in any of the ancient Pacific samples reported here or previously published. Occupation of the Shisanhang site dates to between 2,300 and 500 BP, with most dates clustering around 1,500 to 1,000 BP, and contains evidence for long distance exchange with China^27^. The greatest genetic diversity of modern dogs is found in southeastern China^5,7^, and it is perhaps not unexpected to observe these divergent lineages in a Taiwanese site.

These ancient mitogenomes provide a series of individual insights into the history of dog dispersals from East Asia and their spread eastward across the Pacific, prior to the introgression of modern European dog breeds. Although the sample size is very small, these data from mtDNA sequences from only a few dogs, when combined with archaeological and linguistic data, demonstrate the potential of aDNA studies to differentiate between hypothesised dispersal events. A median-joining network (Fig. 1) shows the relationship between the complete or near complete ancient mitogenomes and modern mitogenomes from dingoes, NGSDs and Southeast Asian village dogs. Bayesian analysis to establish divergence times for the A and B haplogroups provides an estimate of between 3,000 to 21,000 years ago, which is broadly consistent with Duleba and colleagues^19^ findings but overall the tree lacks sufficient resolution to be very informative about such events (Supplementary Fig. S1). The topology of the tree remains the same whether or not dates from archaeological contexts are included, and is consistent with trees generated in MEGA7 using the maximum likelihood method^28^ (Supplementary Fig. S2). A median-joining network of the 582 bp control region haplotypes used previously to investigate dog domestication^5^ and dispersal in the Pacific^8,13^ also demonstrates the close relationship between the dingo haplotypes and the ancient Pacific samples (Supplementary Fig. S3).

## Discussion

### Pre-Lapita introductions

One of the earliest archaeological sites in ISEA with evidence of dogs is Matja Kuru 2 Cave in East Timor, with a dog burial that dates to about 3,000 years ago^29^. Stable isotope analyses indicate that the dog had a diet dominated by terrestrial plant foods, similar to that of Pacific pigs, rather than a diet high in hunted or marine foods. This suggests that the dog may have been associated with a food producing community, rather than one reliant on wild food sources^29^. The mitogenomic sequence we obtained from this specimen (MS10330) carries the A4’5 haplotype (after^19^), which is not shared by any other ancient specimen we sequenced. It is also clearly distinct from the sub-clade of Hg A2b3 where modern sequences from dingoes and NGSDs are located.

The introduction of dingoes to Australia remains enigmatic. Although molecular date estimates have raised the possibility of a late Pleistocene introduction of dogs to Australia^13,18^ there is, as yet, no archaeological evidence for dingoes prior to c. 3,500 years ago. In addition, divergence dates may relate to events that predate the arrival of dingoes to Australia, so have limited application to understanding the introduction timing and process. Recent genetic analyses of modern dingoes and NGSDs show a geographical distribution of dingo mtDNA lineages that distinguishes populations from the northwest and southeast of Australia^18,30^, which we also see in the modern dingo sequences we generated (Fig. 1). This population structure is also observable in results from nuclear and Y-chromosome analyses^30–33^. In addition, the two dingo groups, while more closely related to the NGSDs than to any other dogs, diverge from the NGSD sequence, which may indicate a period of isolation between the two groups of dogs. The only genetic data from NGSDs has been obtained from the captive population derived from only eight dogs^34^. Village dogs in New Guinea however possess many, if not all, of the behavioural and phenotypic characteristics ascribed to NGSDs^35^ and it is possible that sampling from isolated village populations on the New Guinea mainland, and highland NGSD populations if found to still exist, may also be informative about NGSD and dingo ancestral history.

The process of human settlement expansion across the western Torres Strait between New Guinea and Australia may have facilitated the dispersal of dogs/dingoes from New Guinea to Australia. Evidence of this human expansion appears in the archaeological record from around 3,800 years ago, but to date no dog remains have been found from this period^36^. Balme and O’Connor^37^ hypothesise that increasing numbers of small game animals observable in the archaeological record in the mid-Holocene may be indicative of the arrival and use of dingoes by people to assist with hunting. These chronometrically dated changes in faunal composition in archaeological sites, along with molecular evidence indicating at least two introductions to Australia and a close genetic relationship between dingoes and NGSDs, suggest that by the mid to late Holocene deliberate dog translocations were taking place between New Guinea and Australia. Securely dated archaeological material, along with further molecular data, would assist with the refining the date of introductions, routes and possible origins of the dingo.

Interestingly, one sequence we generated from a dog bone excavated from an Iron Age archaeological site in Taiwan, MS10133, is located on the network between these two groups of dingoes (Fig. 1). A similar link to modern Taiwanese dog lineages has been observed in dingo Y-chromosome markers^32^. Sacks and colleagues raise the possibility of a separate dispersal event from Taiwan, or the movement of dogs with Austronesian language speakers both out of China to the south through the Tonkin Gulf and to Taiwan, to explain this observation^32^.

The appearance of the Neolithic in ISEA and its extension out into the western Pacific, characterised by the presence of pottery in archaeological sites dated to between 3,800 and 3,300 years ago, involved the spread of new languages, ideas and identities alongside migration and recruitment from local communities^38^. Molecular analyses of dog remains from MSEA archaeological sites during this critical period of human expansion could greatly assist with tracking the translocations of dogs as part of this movement. What is known archaeologically is that dogs are absent from MSEA hunter gatherer sites. It was only with the expansion of rice and millet farmers into Southeast Asia from Chinese domestication centres that dogs made their appearance about 4,000 year ago, and from this point onwards, dog remains are commonly found. Archaeological excavation at An Song, in Southern Vietnam, has provided dog bones^39^ and at Khok Phanom Di, a central Thai site, dog bones and canid faeces containing rice chaff and fish bones were found^40^. The time period between 4,000 and 3,000 years ago in MSEA and ISEA is central to tracking the movement of dogs across Southeast Asia and out into the islands of Oceania. It is likely that as more samples of dogs from archaeological sites across the region become available, a more detailed picture of the history of ISEA dogs will be revealed.

### Late Lapita dogs

Dogs are often argued to have been moved across the Pacific by people associated with the LCC^4^. However, there is only extremely limited archaeological evidence for dogs in early Lapita archaeological sites, and no dog bone has been reported from Lapita archaeological sites beyond the Bismarck Archipelago (for reviews see^2,41^). The presence of drilled dog teeth in Lapita archaeological sites of Near Oceania, along with the scarcity of other skeletal elements, also indicates that dog remains may have been incorporated into archaeological contexts by mechanisms unrelated to the transport of living dogs, such as the exchange of valuables. Although mtDNA control region sequences indicate a route through ISEA for the ancestors of dogs found in East Polynesian archaeological sites^8,13^, a possible link between Pacific dogs and modern Taiwanese dogs has also been suggested^32^.

Although we were unable to generate complete mitogenomes from the two archaeological specimens from the terminal Lapita and post-Lapita levels at archaeological sites at Edubu 1 (MS10031) and Bogi 1 (MS10023) from Caution Bay on the south coast of mainland Papua New Guinea, we were able to obtain limited coverage of part of their genomes (43% and 17% respectively). Compared to the rest of the ancient mitogenomes we sequenced, these specimens carry diagnostic mutations at several positions indicating that they fall within Hg B (Supplementary Table S3). Sample MS10031 comes from an archaeological context at Edubu 1 that is well dated to between 2,580 and 2,410 cal BP, and is probably closer to the start of this age range^42,43^. This archaeological site is associated with the very end of dentate-stamped ceramics at Caution Bay, that is, it dates to the terminal period of Lapita here as ceramics begin to lose all body decoration (i.e. as all ceramic vessel bodies become plain wares). This is a time of major change in Caution Bay ceramics. Bogi 1 is a multi-phase archaeological site representing an ancient village location. Sample MS10023 was securely embedded within an archaeological context that dates to within the narrow timeframe of 2,150 to 2,100 cal BP^44^. At Caution Bay, dentate-stamped ceramics that define Lapita pottery are commonly found in 2,600 cal BP sites but entirely cease by 2,550 cal BP, indicating that sample MS10023 post-dates Lapita in this area by 400 to 450 years. These two specimens are the first from the Pacific that have been identified with a Hg B lineage and suggest a common ancestor with one of our dog samples from an Iron Age archaeological site in Taiwan and hence a potential link with Taiwan. While this may fit with current linguistic models for the expansion of Austronesian languages from Taiwan, it is also possible, given the limited number of mtDNA sequences available to date, that the B haplogroup could be a shared common ancestral lineage throughout the broader ISEA gene pool. Only further genetic analyses of well-dated dog samples from ISEA will clarify the distribution of B lineages.

### Later introduction of Remote Oceanic dogs

Despite the scarcity of dog remains in Lapita archaeological sites in the western Pacific, there is considerable archaeological evidence for people taking dogs with them during the colonisation of East Polynesia that began around 1,000 years ago^45^. Linguistic evidence, in addition to being used to model human migrations, is also informative about the nature of interactions between people and dogs during the colonisation of the Pacific^46^. Words for items that were a common part of Austronesian, Lapita and Polynesian cultures are transmitted consistently to daughter languages, and are often shared by languages across a wide area. Terms for dog however are not consistently shared across the Pacific and show considerable variability. There is no widely shared cognate for dog in either Proto-Austronesian or Proto-Oceanic (associated with the LCC), and in Oceanic languages the term for ‘dog’ is variable. By the time people were settling the islands of Polynesia the situation appears to have changed. There is a Proto-Polynesian term for dog, *kuli, which alongside the terms for chicken and pig, is a unique innovation. These terms first appear in Proto-Polynesian, and regular cognates consistently appear in daughter languages, including the Polynesian Outlier languages to the west^47^. This contrast suggests that, by the time that the colonisation of East Polynesia commenced, the Polynesians had developed linguistic and cultural innovations that included a highly successful method of transport and establishment of domestic animals not seen previously in the region^46^.

The complete and near-complete ancient mitogenomes we sequenced from dogs from archaeological sites from Atafu, an atoll of Tokelau in West Polynesia, and Hawai’i in East Polynesia, and three previously published sequences from New Zealand^17^ and the southern Cook Islands^26^, form a haplogroup A2b2 which is separate from dingoes and NGSDs (Fig. 1). A partial sequence from a dog burial from Graciosa Bay on Santa Cruz in the Solomon Islands also has markers consistent with this haplogroup. Ancient mitogenomes from dog specimens from the Taurama archaeological site near Port Moresby in Papua New Guinea and Goemu on Mabuyag island in the Torres Strait are also part of this haplogroup, which is distinct from the B lineage observed in the terminal Lapita and post-Lapita dogs from earlier archaeological sites along the south coast of mainland Papua New Guinea, or the dingo and NGSD group. This Hg A2b2 comprises two sub-clades, with haplotypes from Near Oceania and Polynesia found in both clades (Supplementary Table S1, Supplementary Figs S1 and S2).

The Taurama archaeological site dates to between 2,000 and 1,200 BP, where a dog burial was found in an archaeological context associated with Early Papuan Pottery (EPP)^48^. EPP archaeological sites begin to appear along the south coast of mainland Papua New Guinea around 2,000 BP and provide evidence of a period of cultural change, including new pottery designs^49^. The mtDNA sequence from the Taurama dog bone (MS10329) is the earliest known Oceanic occurrence of Hg A2b2* and may indicate the arrival of dogs from a different source population to those in the terminal and post-Lapita Caution Bay archaeological sites.

The appearance of this new A2b2* genetic lineage may be linked to a notable increase in the numbers of dog bone in Near Oceania and the sudden appearance of dog bones in West Polynesian archaeological sites beginning around 2,000 years ago^46^, suggesting there may have been a significant change in relationships between dogs and people in the Pacific at this time. We have previously suggested that this may signal changes in technology and animal husbandry that facilitated the successful movement of dogs further into the Pacific than ever before, possibly also linked with the impetus for the initial colonisation of East Polynesia^46^. Other possibilities involve a movement of new people and their dogs into Polynesia^50^ or the incorporation of dogs into existing cultures via exchange networks. The presence of this A2b2* lineage in Near Oceania may also be associated with a westwards (as well as eastwards) expansion of Remote Oceanic groups resulting in the establishment of the Polynesian Outliers in the western Pacific.

The ancient sequences from the major and widespread A2b2* sub-clade are most similar to partial sequences we obtained from two dog specimens from Bronze Age archaeological sites in Thailand (MS10331 and MS10332). Although we were unable to obtain complete mitogenomes from the archaeological Thai specimens, we were able to examine enough nucleotide positions with adequate coverage (covered by at least three reads) to indicate that they are consistent with the A2 sub-clade^19^. Compared to the wider dataset of modern dog mitogenomes, the ancient Pacific dog mitogenomes are part of the A2b2* sub-clade^19^, also shared with modern dogs sampled in Thailand^5^, Indonesia^31^ and Fiji^19^. This indicates that dogs from Hg A2b2* in the Pacific may have ultimately been derived from MSEA dogs, rather than being incorporated into Austronesian expansions southward from Taiwan. High frequencies of two short control region haplotypes, found in ancient Polynesian dogs, have also been observed in modern Indonesian dogs^13^. A similar southwestern route through ISEA has been suggested for the dispersal of pigs^51^. It is possible that some modern village dogs in ISEA carry ancient lineages associated with the dispersal of dogs into the Pacific, but as yet there is very little comparative data for dog mitogenomes in that region. Shannon and colleagues^52^ surveyed autosomal, mitochondrial and Y-chromosome diversity in modern purebred and village dogs. They found that while some populations of village dogs are almost entirely descended from European dogs, especially in the neotropics and Remote Oceania, others show little evidence of admixture. Notably, village dog populations sampled from Papua New Guinea and the Solomon Islands comprise a mix of indigenous and European ancestry, while those from Borneo have no observable European ancestry and are similar to Vietnamese dogs. The Vietnamese dogs sampled also showed little evidence of European admixture. These results suggest that studies of modern village dogs in MSEA and ISEA may be especially valuable in advancing our understanding of dog dispersals through Southeast Asia into Oceania, particularly where aDNA data are unavailable.

## Conclusion

The dispersal of dogs across the Pacific is inseparably linked to the relationships between dogs and people. Unlike movement across continental landmasses, Pacific dogs must have been transported by people across the waters that separate islands. The ancient mitogenomes sequenced from archaeological dog specimens presented here offer a novel series of individual insights into the history of dog translocation from Southeast Asia as it occurred prior to the influence of modern European dog breeds. We generated seven mitogenomes and five partial sequences from ancient MSEA, ISEA and Pacific dogs, and four modern dingoes. Despite the small sample size, our results reveal levels of complexity and discontinuity in the introduction and movement of dogs, which are mirrored in the archaeological and linguistic evidence, suggesting at least three introductions of dogs to the wider Pacific region, in addition to the earlier appearance of the dingo in Australia. Further mtDNA studies of ancient dogs and modern village populations throughout the region may contribute additional data that can be used to evaluate these hypothesised dispersals. Autosomal and Y-chromosome analyses also have the potential to generate additional information about dog dispersal, which could reveal different dispersal signatures based on sex, or phenotypic characteristics, though the environmental conditions in the region are not particularly conducive to aDNA preservation.

Our molecular genetic analyses reveal one of the earliest dogs present in ISEA around 3,000 years ago from Timor-Leste possesses a mtDNA lineage not found elsewhere in the region. We also found similarities between mtDNA of modern dingoes and NGSDs and an ancient Taiwanese sequence, which supports previous observations about possible links between Y-chromosome markers of modern dingoes and a modern Taiwanese sample^32^. More work is required to address whether these connections reflect the genetic diversity of a shared ancestral population in mainland China, or attest to a currently unknown dispersal event linking the two populations. Archaeological evidence for the introduction of dogs to Oceania as part of the LCC is extremely limited. Nonetheless, we demonstrate that mitogenomes from dogs in terminal Lapita and post-Lapita levels of archaeological sites along the south coast of mainland New Guinea also show affinities with an Iron Age dog specimen from Taiwan, raising the possibility of at least one introduction of dogs during Austronesian expansions ultimately from the north. Finally, we have identified a major late introduction of dogs across the islands of Oceania beginning around 2,000 years ago, which appears to have originated in MSEA, not Taiwan, and culminated in the establishment of dog populations in initial colonisation-era sites throughout East Polynesia.

## Materials and Methods

### Ancient dna extraction, library preparation, sequencing and data processing

Twelve dog specimens comprising bone and teeth from thirteen archaeological sites were used for this study (Supplementary Table S1), in conjunction with three ancient specimens previously published (Supplementary Table S2)^17,26^. The mitogenomes reported here were generated using hybridisation capture and were sequenced on the Illumina MiSeq platform as previously described^17^, in accordance with protocols to ensure the authenticity of aDNA sequences. Throughout DNA extraction and library preparation, blank extractions were processed alongside samples to provide negative controls.

We processed the raw sequence reads using a computational protocol previously described^17^. A notable feature of this pipeline is the use of a composite mitochondrial reference genome (made up of likely contaminant genomes such as pig, cow, chicken and human) to check for contamination (Supplementary Table S4). Coverage and read depth plots were created for all samples (Supplementary Figs S4 and S5). All reads displayed the expected characteristics of aDNA, including fragment length and deamination patterns (Supplementary Fig. S6). To maximize sequence usability in downstream analyses, imputation was performed and sites with greater than 80% confidence were retained. Consensus sequences containing insertions and/or deletions (indels) were produced for each sample that passed quality control, and these were deposited in Genbank (accession numbers KT168373, KY98508–KY98516). In these Genbank sequences non-variant sites that were supported by fewer than three reads were changed to ‘Ns’.

### Modern dingo mitogenomes

Blood samples from four dingoes from Wellington Zoo were collected by the Veterinary Science Manager, Dr Lisa Argilla and sent to the University of Otago. DNA was extracted using the MagJET magnetic bead (ThermoFisher) protocol (as per manufacturer’s instructions). Four long-range primer pairs were used to amplify the complete mitogenome (Supplementary Text). Blunt end repair, ligation of sequencing adaptors and barcoding were carried out following^53^, with modifications for Illumina sequencing adaptors. Sequencing was carried out on the Illumina MiSeq platform. Reads were processed using a modified version of the ancient DNA pipeline previously described^17^.

### Phylogenetic reconstructions

The phylogenetic structure of the archaeological dog samples, modern dingoes and published modern dog sequences used in this study (Supplementary Tables S1 and S2) was characterised using network analysis Popart v1.7.1^54^. Imputed VCF files were converted to FASTA and then to NEXUS format, using a custom python script and Biopython^55^ respectively. This dataset was then used to create a median-joining network using Popart with default settings (Fig. 1). We used jmodeltest2 (v2.1.7)^56^ to assess different models of evolution for the ancient and modern dog sequences. The best model was then selected based on the Bayesian information criterion (BIC). According to this criterion a HKY + I model was most appropriate. Using this model, a maximum likelihood (ML) tree was generated using MEGA7^28^ with 10,000 bootstrap replicates (Supplementary Fig. S2). Bayesian trees were created with BEAST (v1.8.2)^57^ using the Birth-Death tree model and 10,000,000 generations, and sampling every 1000th generation (Supplementary Fig. S1). The maximum credibility tree was determined using TreeAnnotator with 25% of the trees being discarded as burn-in. Convergence diagnostics were investigated using Tracer (v1.4.0) and the phylogenetic tree was visualised in FigTree (v1.4.0).

To compare the newly sequenced Pacific dog sequences with published data, the 582 bp control region fragment was aligned with control region fragments available in GenBank, using mafft v7^58^. This dataset comprised a total of 408 sequences and was used to create a median-joining network using Popart v1.7.1^54^ (Supplementary Fig. S3).

### Data availability statement

Consensus sequences are available from the GenBank database (accession numbers KY798508-KY798516, MH035673-MH035676).

## Electronic supplementary material

Supplementary Information
