# Supplementary material for: Complex history of dog (Canis familiaris) origins and translocations in the Pacific revealed by ancient mitogenomes
Source: Sci Rep. 2018 Jun 14;8:9130. doi: 10.1038/s41598-018-27363-8 (PMC6002536; doi:10.1038/s41598-018-27363-8)
Supplement: Supplementary file 1 — Supplementary Information [file 41598_2018_27363_MOESM1_ESM.pdf]

Supplementary Information:

Complex history of dog (*Canis familiaris*) origins and translocations in the Pacific revealed by ancient mitogenomes

Greig, K., Gosling, A., Collins, C.J., Boocock, J., McDonald, K., Addison, D. J., Allen, M. S., David, B., Gibbs, M., Higham, C. F.W., Liu, F., McNiven, I.J., O'Connor, S., Tsang, C. H., Walter, R., & Matisoo-Smith, E.

Supplementary Tables

Table S1. Ancient Mainland, Island South East Asian and Pacific complete mitogenomes and partial sequences.

1A. Complete ancient mitogenomes Laboratory specimen numbers, GenBank accession number, mitogenome coverage (% of bases), read depth statistical summary and imputed bases (%), and archaeological context (site name, location, age).

Specimen ID.	***GenBank***	***Coverage (%)***	***Mean read depth***	***Standard deviation of read depth***	***Haplogroup (after Duleba and colleagues[1])***<br>***Sub-clade indicated by bracketed letter.***	***Imputed variable sites (%)***	***Archaeological site and location***	***Date (source of date)***
MS10016	KY798516	98.33	6.987505231	3.360716471	A2b2(a)	0.094936709	Fale Islet (TU-2), Atafu, Tokelau	500-310 cal. BP (at 2σ)[2]
MS10043	KY798514	98.45	10.34435344	7.609122939	A2b2(b)	0.088607595	Goemu, Mabuyag Island, Torres Strait	950 - 1000 BP[3]
MS10329	KY798515	90.5	3.725473785	3.237475759	A2b2(b)	0.436708861	Taurama, Papua New Guinea	1000 - 2000 BP[4]
MS10330	KY798511	98.19	6.897351587	3.166819041	A4’5	0.094936709	Matja Kuru 2, East Timor	2921–3075 cal. BP (Calib 6.1.0, two sigma, p=0.95)[5]
MS10333	KY798512	96.71	5.698810307	2.920348464	A2b3	0.082278481	Shisanhang (SSH), Taiwan	ca. 1800-500 years BP[6]

MS10334	KY798513	99.97	36.19824236	17.7893809	B	0	Shisanhang (SSH), Taiwan	ca. 1800-500 years BP[6]
MS10665	KY798508	100	152.9559993	56.13345546	A2b2(b)	0	Nu'alolo Kai, Kauai, Hawai'i	1600-1800 AD[7]
1B. Partial ancient sequences. Laboratory specimen numbers, mitogenome coverage (% of bases) read depth statistical summary and imputed bases (%), archaeological context (site name, location, age).								
MS10023		43.06	0.590960722	0.826636889	B*	N/A	Bogi 1, South Coast, Papua New Guinea	2150-2100 cal BP[8].
MS10031		17.20	0.191606385	0.44289477	B*	N/A	Edubu 1, South Coast, Papua New Guinea	2580-2410 cal BP[9]
MS10090		83.71	2.127697734	1.645368195	A2b2*	N/A	Nembo, Graciosa Bay, Santa Cruz, Solomon Islands	Late 16th century[11]
MS10331		66.2	1.093860226	1.076768681	A2*	N/A	Ban Chiang, Thailand	1608 and 1442 cal. BCE[12]
MS10332		79.36	2.247205117	1.97359114	A2*	N/A	Nong Nor, Thailand	1000 -700 BC Bronze Age[12]

Table S2: Published ancient and modern sequences used in this study.

**GenBank No.**	**Sample, Type and Location**	**Age**	**Source of sequence**
KT168373	MS10062, Dog, Wairau Bar, New Zealand	Ancient	Previous study[13]
KY798510	MS10326, Dog, Ureia (AIT-10), Zone G, Aitutaki, Cook Islands.	Ancient	Previous study[14]
KY798509	MS10327, Dog, Ureia (AIT-10), Zone E, Aitutaki, Cook Islands	Ancient	Previous study[14]
MH035674	DB, Dingo, Wellington Zoo, New Zealand	Modern	This study
MH035673	DK, Dingo, Wellington Zoo, New Zealand	Modern	This study
MH035675	DY, Dingo, Wellington Zoo, New Zealand	Modern	This study
MH035676	DW, Dingo, Wellington Zoo, New Zealand	Modern	This study
JX088671	Dingo, Simpson Desert, Australia	Modern	GenBank
JX088672	Dingo, Gibson Desert, Australia	Modern	GenBank
JX088673	Dingo, Gibson Desert, Australia	Modern	GenBank
JX088674	New Guinea Singing Dog	Modern	GenBank
JX088675	Dingo, Gibson Desert, Australia	Modern	GenBank
JX088676	Dingo, Fraser Island, Australia	Modern	GenBank
JX088677	Dingo, Simpson Desert, Australia	Modern	GenBank
JX088678	Dingo, Simpson Desert, Australia	Modern	GenBank
JX088679	Dingo, Alpine, Australia	Modern	GenBank
JX088680	Dingo, Alpine, Australia	Modern	GenBank
JX088681	Dingo, Kimberley, Australia	Modern	GenBank
JX088682	Dingo, Kimberley, Australia	Modern	GenBank
JX088683	Dingo, Kimberley, Australia	Modern	GenBank
JX088684	Dingo, Kimberley, Australia	Modern	GenBank

JX088685	Dingo, Gibson Desert, Australia	Modern	GenBank
JX088686	Dingo, Simpson Desert, Australia	Modern	GenBank
JX088687	Dingo, Gibson Desert, Australia	Modern	GenBank
JX088688	Dingo, Alpine, Australia	Modern	GenBank
JX088689	Kalimantan village dog, ISEA	Modern	GenBank
JX088690	Bali village dog, ISEA	Modern	GenBank
JX088691	Dingo, Kimberley, Australia	Modern	GenBank
JX088692	Dingo, Alpine, Australia	Modern	GenBank
JX088693	Dingo, Simpson Desert, Australia	Modern	GenBank
EU789737	A18_m257, Thai village dog, MSEA	Modern	[15]
EU789677	A119_m288, Thai village dog, MSEA	Modern	[15]
EU789736	A18_m261, Thai village dog, MSEA	Modern	[15]
EU789673	A75_m286, Thai village dog, MSEA	Modern	[15]
EU789664	A65_m276, Thai village dog, MSEA	Modern	[15]
EU789682	A122_m266, Thai village dog, MSEA	Modern	[15]
EU789719	A18_m260, Thai village dog, MSEA	Modern	[15]
KF661037	Dingo, Australia	Modern	[16]

Table S3: Variable positions used for haplogroup assignment (after [1]).

	MS10062	MS10326	MS10327	MS10016	MS10090	MS10043	MS10329	MS10330	MS10331	MS10332	MS10333	MS10334
Informative positions	Wairau Bar	Aitutaki	Aitutaki	Tokelau	Santa Cruz	Torres Strait	Taurama	East Timor	Thailand	Thailand	SSH	SSH
		32T										
		54T										
		82T										
												381A
								445G	445G			445G
											642T	
	833G	833G	833G	833G	833G	833G	833G					
								1134G				
								1196A	1196A			
	1454A		1454A	1454A	1454A	1454A	1454A				1454A	1454A
		1725A										
												1756T
												2185C
								2190G	2190G			
								2232G				2232G
							2636A					
	2678.1 G	2678.1 G	2678.1 G	2678.1 G		2678.1 G		2678.1 G	2678.1 G	2678.1 G	2678.1 G	2678.1 G
	2683A	2683A	2683A	2683A	2683A	2683A	2683A	2683A	2683A	2683A	2683A	2683A
										2800T	2800T	
												2812T
	2962T	2962T	2962T	2962T	2962T	2962T	2962T	2962T	2962T	2962T	2962T	2962T
			3028C	3028C				3028C				3028C
	3196C	3196C	3196C	3196C	3196C	3196C	3196C	3196C	3196C	3196C	3196C	3196C
								3238A				
			3257T									
	3406T	3406T	3406T	3406T	3406T	3406T	3406T	3406T	3406T			3406T

												3469A
												3598A
								4204A	4204A			4204A
										4277G		4277G
												4390C
											4466A	
												4517A
			4646C									4646C
												5009T
	5107A	5107A		5107A		5107A						
	5126T	5126T	5126T	5126T	5126T	5126T	5126T				5126T	
	5367T	5367T	5367T	5367T	5367T	5367T	5367T	5367T	5367T	5367T	5367T	5367T
	5444C	5444C	5444C	5444C	5444C	5444C	5444C	5444C	5444C	5444C	5444C	5444C
	5549T	5549T		5549T	5549T	5549T	5549T					
			6009A									
			6027A									
	6065G	6065G	6065G	6065G	6065G	6065G	6065G	6065G	6065G	6065G	6065G	6065G
												6257A
												6470A
								6653G				
			6764T									6764T
	6854T	6854T			6854T	6854T						
												7014C
	7914G	7914G	7914G	7914G	7914G	7914G	7914G				7914G	
											7994A	
	8048G	8048G		8048G	8048G	8048G	8048G				8048G	
												8101A
												8221C
												8225C
	8281C	8281C	8281C	8281C	8281C	8281C	8281C	8281C	8281C		8281C	8281C
	8323G	8323G	8323G	8323G	8323G	8323G	8323G			8323G	8323G	8323G
	8368T	8368T	8368T	8368T	8368T	8368T	8368T	8368T	8368T	8368T	8368T	8368T
	8411C	8411C		8411C		8411C	8411C					
												8425A
			8569G									8569G
	8612T	8612T	8612T	8612T	8612T	8612T	8612T					
	8703A	8703A	8703A	8703A	8703A	8703A	8703A	8703A	8703A	8703A	8703A	8703A
												8736C
												8760G

	8764T	8764T	8764T	8764T	8764T	8764T	8764T	8764T	8764T	8764T	8764T	8764T
	8807A	8807A	8807A	8807A	8807A	8807A	8807A	8807A	8807A	8807A	8807A	8807A
												8817G
	8877G	8877G		8877G	8877G	8877G						8877G
			8904T									
										8926A	8926A	
								8991G	8991G			8991G
								9189C				
												9219G
	9357C	9357C	9357C	9357C	9357C	9357C	9357C					
							9684A					
								9756G				
											9757T	
												9825A
	9911.1<br>T	9911.1<br>T	9911.1<br>T	9911.1<br>T	9911.1<br>T	9911.1<br>T	9911.1<br>T	9911.1<br>T	9911.1<br>T	9911.1<br>T	9911.1<br>T	9911.1<br>T
	9911.2<br>G	9911.2<br>G	9911.2<br>G	9911.2<br>G	9911.2<br>G	9911.2<br>G	9911.2<br>G	9911.2<br>G	9911.2<br>G	9911.2<br>G	9911.2<br>G	9911.2<br>G
												10257A
	10354C	10354C	10354C	10354C	10354C	10354C					10354C	
								10404T				10404T
												10440C
											10503C	
								10512A				
							10533T					10533T
							10542<br>G					10542<br>G
												10773C
	10917A	10917A	10917A	10917A	10917A	10917A	10917A	10917A	10917A	10917A	10917A	10917A
	10992A	10992A		10992A		10992A	10992A	10992A	10992A	10992A	10992A	10992A
							11138T				11138T	
	11157C	11157C	11157C	11157C		11157C						
									11176T	11176T		11176T
									11250C	11250C		11250C
							11271T					
												11402C
					11680C							
												11948<br>G
												11963T
								12545<br>G				
									12665C	12665C		12665C
	12788C	12788C	12788C	12788C	12788C	12788C	12788C	12788C	12788C	12788C	12788C	12788C
			12813A									12813A

	13031 G	13031 G	13031 G	13031 G	13031 G	13031 G	13031 G		13031 G	13031 G		
							13065A					
	13202C	13202C	13202C	13202C	13202C	13202C	13202C		13202C	13202C	13202C	
				13261T								13261T
	13299A	13299A	13299A	13299A	13299A	13299A	13299A	13299A			13299A	13299A
					13302T							
								13594A	13594A			13594A
								13618 G	13618 G			13618 G
												13660T
									13708T			13708T
									13777A			13777A
	13864T			13864T	13864T	13864T						
	14329 G	14329 G	14329 G	14329 G	14329 G	14329 G	14329 G		14329 G	14329 G	14329 G	
	14383 G	14383 G	14383 G	14383 G	14383 G	14383 G	14383 G		14383 G	14383 G	14383 G	
											14467T	
											14500T	
											14650T	
												14671A
			14711T									
								14927T				
												14930C
	14998 G	14998 G	14998 G	14998 G	14998 G	14998 G	14998 G		14998 G	14998 G	14998 G	
	15172T	15172T	15172T	15172T	15172T	15172T	15172T			15172T		
	15214A	15214A	15214A	15214A	15214A	15214A	15214A	15214A	15214A	15214A	15214A	15214A
								15316T				
												15435A
												15526T
												15595T
												15612C
	15627 G	15627 G	15627 G	15627 G	15627 G	15627 G	15627 G	15627 G	15627 G	15627 G	15627 G	
												15632T
	15639A		15639A		15639A	15639A		15639A	15639A	15639A	15639A	
												15639 G
												15643 G
	15645 G		15645 G		15645 G	15645 G						
	15652A		15652A	15652A	15652A	15652A			15652A	15652A	15652A	15652A
												15800C
	15814T	15814T	15814T	15814T	15814T	15814T	15814T	15814T	15814T	15814T	15814T	15814T

												15815C
												15912T
								15955T				15955T
												16003 G
								16025C				
								16033 G				

Table S4: Defining SNPs for Haplogroups B and A (after [1]) observed in ancient sequences from dog remains excavated from terminal and post-Lapita archaeological sites on the South Coast, Papua New Guinea.

Position in dog reference genome NC_02008 (16)		MS10023		MS10031	
		Read depth	Nucleotide	Read depth	Nucleotide
**Defining SNPS for BCE haplogroup**					
16	C	-		-	
381	A	-		X1	A
8221	C	X1	C	X1	C
8760	G	-		-	
8877	G	-		-	
13618	G	X3	2/3 G	X1	G
15955	T	X1	T	-	
16003	G	-		-	
**Defining SNPS for BE haplogroup**					
2812	T	-		-	
4277	G	X1	0/1 G	X1	G
4390	C	-		X1	C
8569	G	X1	G	X1	C
8736	C	-	-	-	
8817	G	X1	G	-X1	A
10440	C	X1		-	
12665	C	X1	C	-	
14671	A	X1	A	X1	A
15632	T	-		-	
15652	A	-		-	
**Defining SNPS for B haplogroup**					
2185	C	X1	C	-	
4204	A	-		X1	A
6764	T	-		-	
11948	G	-		-	
13594	A	X2	A	X2	1/2 A
15595	T	-		-	
15612	C	-		-	
15639	G	-		-	
15643	G	-		-	
15815	C	X3	1/3 C	-	
**Defining SNPS for B1 haplogroup**					
445	G	X1	G	X1	G
4646	C	X1	C	-	
5219	G	X1	0/1	-	
9825	A	-		-	
10257	A	-		X1	G
10542	G	X1	G	-	
14608	A	-		-	
14930	C	X2	C	-	

Position in dog reference genome NC_02008[15]		MS10023		MS10031	
		Read depth	Nucleotide base called if different to 'A' SNP	Read depth	Nucleotide
**Defining SNPS for A haplogroup**					
1454	G	-		-	
3469	G	x1	0/1 (A)	-	
3598	G	-			
4517	G	X1	0/1 (A)	-	
5009	T	-		-	
6257	G	-		-	
6470	G	-		-	
8225	T	X1	0/1 (C)	-	
8425	G	-		-	
8991	A	X3	0/3 (2x G)	X1	0/1 (G)
10404	C	-		-	
10533	A	X2	0/2 (T)	X1	0/1 (T)
11250	T	X1	0/1 (T)	-	
11402	T	X1	0/1 (C)	-	
11936	C	-		-	
12813	G	X1	0/1 (A)	-	
13261	C	X2	0/2 (T)	-	
13660	C	X1	1/1 C	-	
13708	C	X1	0/1 T	-	
13777	T	-		-	
15435	G	-		-	
15639	T	-		-	
15800	T	X4	2/4 (C)	-	
15912	C	-		-	

Table S5: Levels of contamination observed in the ancient samples

**Sample ID**	**Human (gi|251831106|ref|NC_012920.1|)**	**Chicken (gi|5834843|ref|NC_001323.1|)**	**Cow (gi|60101824|ref|NC_006853.1|)**	**Pig (gi|223976078|ref|NC_012095.1|)**	**Rat (gi|225622216|ref|NC_012389.1|)**
MS10016	0.180027174	0	0.122961957	0.180027174	0
MS10023	0.695652174	0	0	0.695652174	0
MS10031	3.607142857	0.107142857	0.035714286	3.607142857	0.017857143
MS10043	0.142501246	0	0	0.142501246	0
MS10062	0	0	0.000406959	0	5.09E-05
MS10090	0.119834711	0	0	0.119834711	0
MS10665	0.000848794	0	0.015945192	6.06E-05	0
MS10326	0.135817308	0	0	0.135817308	0
MS10327	0.11040146	0	0	0.11040146	0
MS10329	0.215817694	0	0	0.215817694	0
MS10330	0.495862069	0.026206897	0.008965517	0.495862069	0.006206897
MS10331	2.19245283	0.018867925	0.018867925	2.19245283	0.018867925
MS10332	0.178970917	0	0	0.178970917	0
MS10333	0.057396928	0	0.001616815	0.057396928	0
MS10334	0.02184466	0.000110327	0.067409532	0.02184466	0.001213592

References

1 Duleba, A., Skonieczna, K., Bogdanowicz, W., Malyarchuk, B. & Grzybowski, T. Complete mitochondrial genome database and standardized classification system for Canis lupus familiaris. *Forensic Science International: Genetics* 19, 123-129, doi:10.1016/j.fsigen.2015.06.014 (2015).

2 Addison, D. J. *et al.* Archaeology of Atafu, Tokelau: Some initial results from 2008. *Rapa Nui Journal* 23, 5-9 (2009).

3 McNiven, I. J. *et al.* Midden formation and marine specialisation at Goemu village, Mabuyag, Torres Strait, before and after European contact. *Memoirs of the Queensland Museum, Culture* 8, 377 (2015).

4 Matisoo-Smith, E. in *Genes, language and culture history in the Southwest Pacific* (ed J S Friedlaender) 157-170 (Oxford University Press, 2007).

5 Gonzalez, A., Clark, G., O'Connor, S. & Matisoo-Smith, L. A 3000 year old dog burial in Timor-Leste. *Australian Archaeology* 76, 13-20 (2013).

6 Tsang, C. Recent advances in the Iron Age archaeology of Taiwan. *Bulletin of the Indo-Pacific Prehistory Association* 20, 153-158 (2000).

7 Graves, M., Field, J. & McElroy, W. An overview of site 50-30-01-196, Nu ʻalolo Kai, Kaua ʻi: Features, excavation, stratigraphy, and chronology of historic and prehistoric occupation. *Na Mea Kahiko o Kaua ʻi: Archaeological studies in Kaua ʻi. Special Publication*, 149-187 (2005).

8 David, B. *et al.* A new ceramic assemblage from Caution Bay, south coast of mainland PNG: the linear shell edge-impressed tradition from Bogi 1. *Journal of Pacific Archaeology* 3, 73-89 (2012).

9 McNiven, I.J. et al. In Peopled Landscapes: Archaeological and Biogeographic Approaches to Landscapes (eds SG Haberle and B David) 121-156 (Terra Australis, 2012).

10 Petchey, F. *et al.* 14C marine reservoir variability in herbivores and deposit-feeding gastropods from an open coastline, Papua New Guinea. *Radiocarbon* 54, 967-978 (2012).

11 Gibbs, M., Duncan, B. & Kiko, L. Spanish Maritime Exploration in the South-west Pacific: the search for Mendaña's lost almiranta, Santa Isabel, 1595. *International Journal of Nautical Archaeology* 44, 430-438 (2015).

12 Higham, C. *et al.* The Origins of the Bronze Age of Southeast Asia. *Journal of World Prehistory* 24, 227-274, doi:10.1007/s10963-011-9054-6 (2011).

13 Greig, K. *et al.* Complete Mitochondrial Genomes of New Zealand's First Dogs. *PLoS One* 10, e0138536, doi:10.1371/journal.pone.0138536 (2015).

14 Greig, K., Boocock, J., Allen, M. S., Walter, R. & Matisoo-Smith, E. Ancient DNA evidence for the introduction and dispersal of dogs in New Zealand. *Journal of Pacific Archaeology* 9, 1-10 (2018).

15 Pang, J. F. *et al.* mtDNA Data Indicate a Single Origin for Dogs South of Yangtze River, Less Than 16,300 Years Ago, from Numerous Wolves. *Molecular Biology and Evolution* 26, 2849-2864, doi:10.1093/molbev/msp195 (2009).

16 Thalmann, O. *et al.* Complete Mitochondrial Genomes of Ancient Canids Suggest a European Origin of Domestic Dogs. *Science* 342, 871-874, doi:10.1126/science.1243650 (2013).

Supplementary Figures

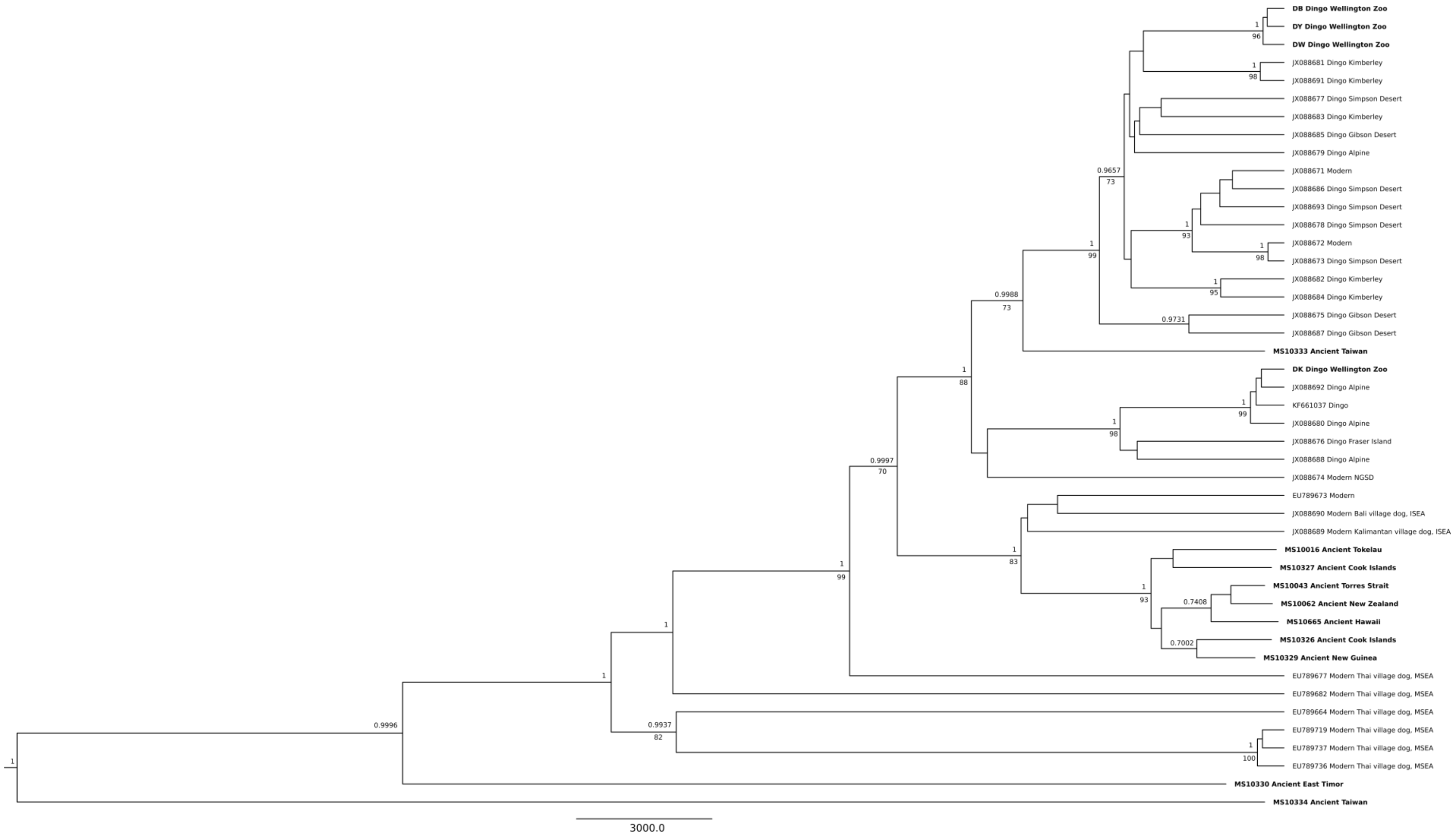

Figure S1: Bayesian tree based on complete mitogenome (16774 bp) of ancient Pacific, ISEA and MSEA dogs, modern dingoes and modern Southeast Asian dogs (Tables S1 and S2). Posterior probabilities (>0.7) are shown above phylogenetic nodes and bootstrap values (>70), based on Maximum Likelihood analysis, are shown below nodes. Scale bar represents time in years.

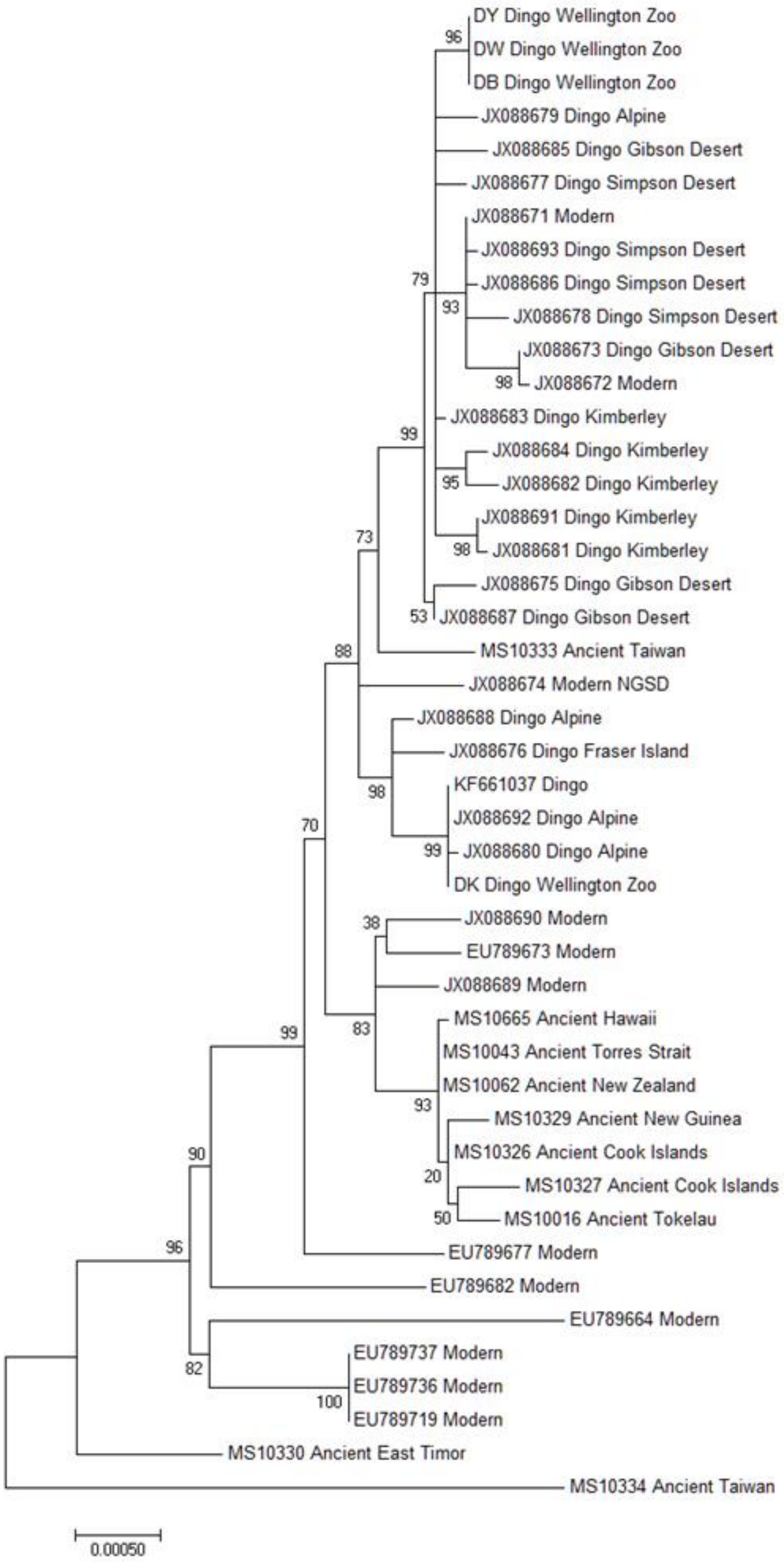

Figure S2: Molecular phylogenetic analysis by maximum likelihood method, implemented in MEGA7[1]. The evolutionary history shown inferred by using the maximum likelihood method based on the Hasegawa-Kishino-Yano model. The tree with the highest log likelihood (-25257.5243) is shown. The percentage of trees in which the associated taxa clustered together is shown next to the

branches. Initial tree(s) for the heuristic search were obtained automatically by applying Neighbor-Join and BioNJ algorithms to a matrix of pairwise distances estimated using the Maximum Composite Likelihood (MCL) approach, and then selecting the topology with superior log likelihood value. A discrete Gamma distribution was used to model evolutionary rate differences among sites (5 categories (+G, parameter = 0.0500)). The rate variation model allowed for some sites to be evolutionarily invariable ([+I], 0.0010% sites). The tree is drawn to scale, with branch lengths measured in the number of substitutions per site. The analysis involved 45 nucleotide sequences. There were a total of 16774 positions in the final dataset.

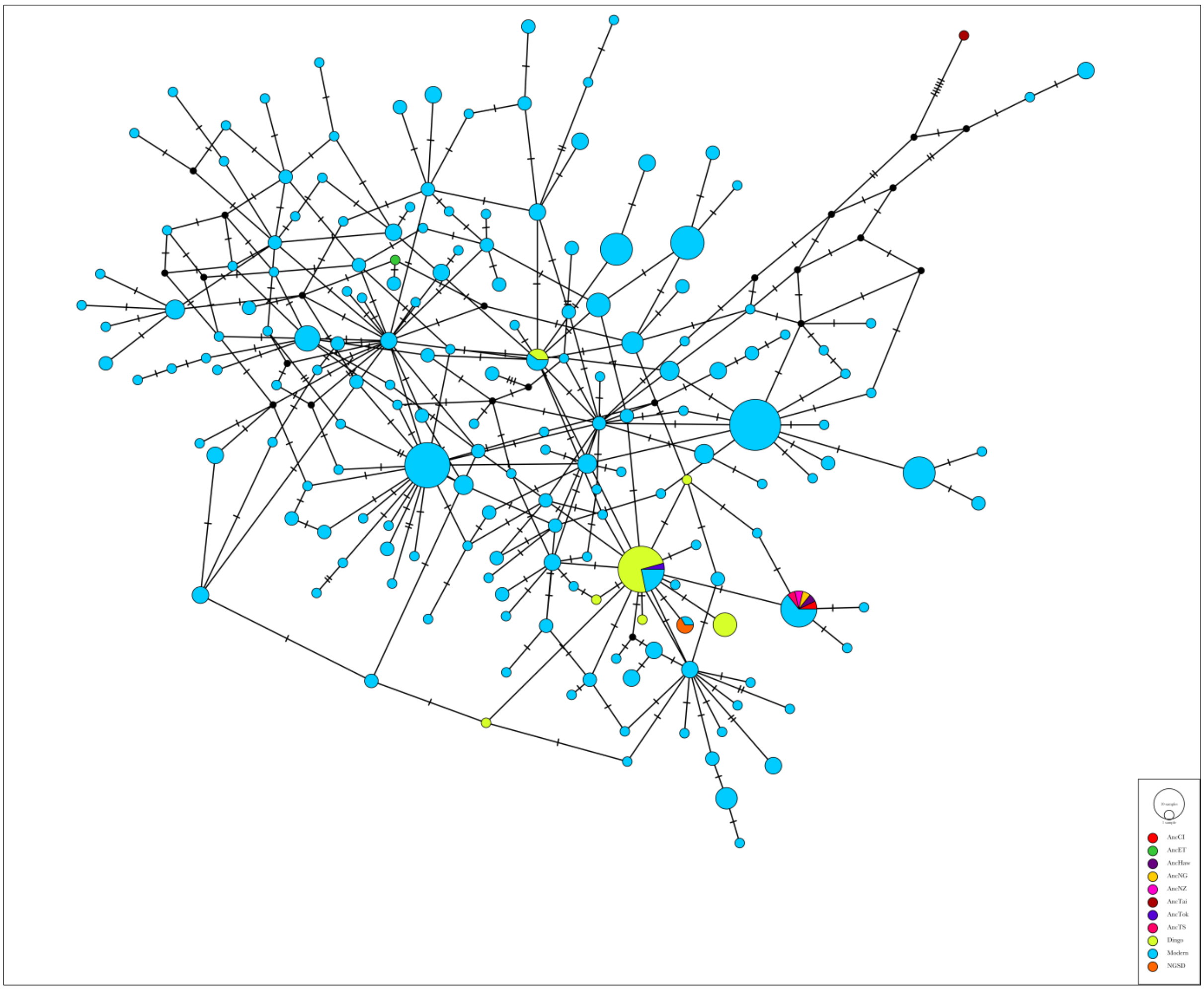

Figure S3: Median-joining network of 582 bp control region fragment (after [2]) constructed using Popart v1.7.1[3].

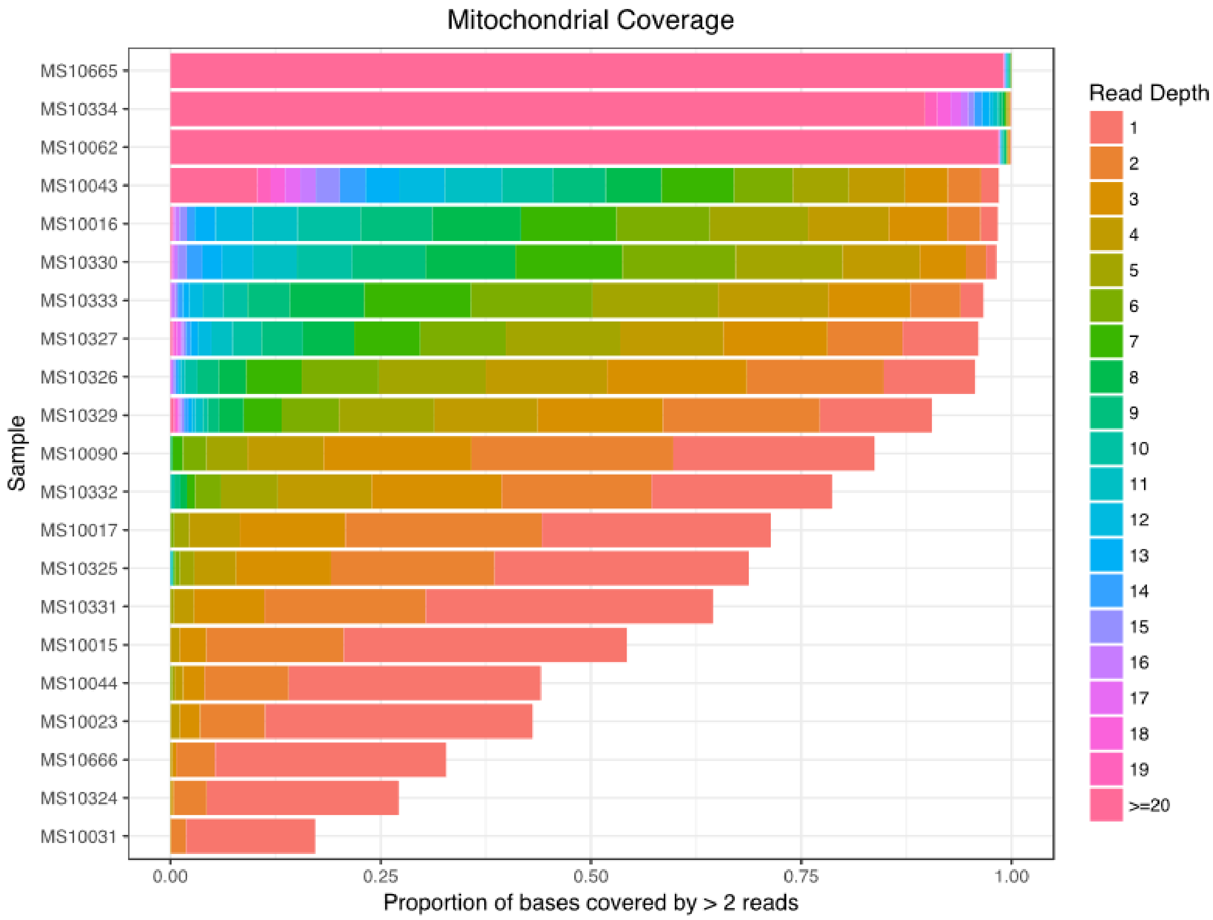

Figure S4: Graph of mitogenome coverage of the ancient dog sequences. Each sample is represented by a horizontal bar. The length of each bar on the x-axis shows the proportion of the mitochondrial genome covered by at least one read. The read depth is shown by the colour gradient given in the legend.

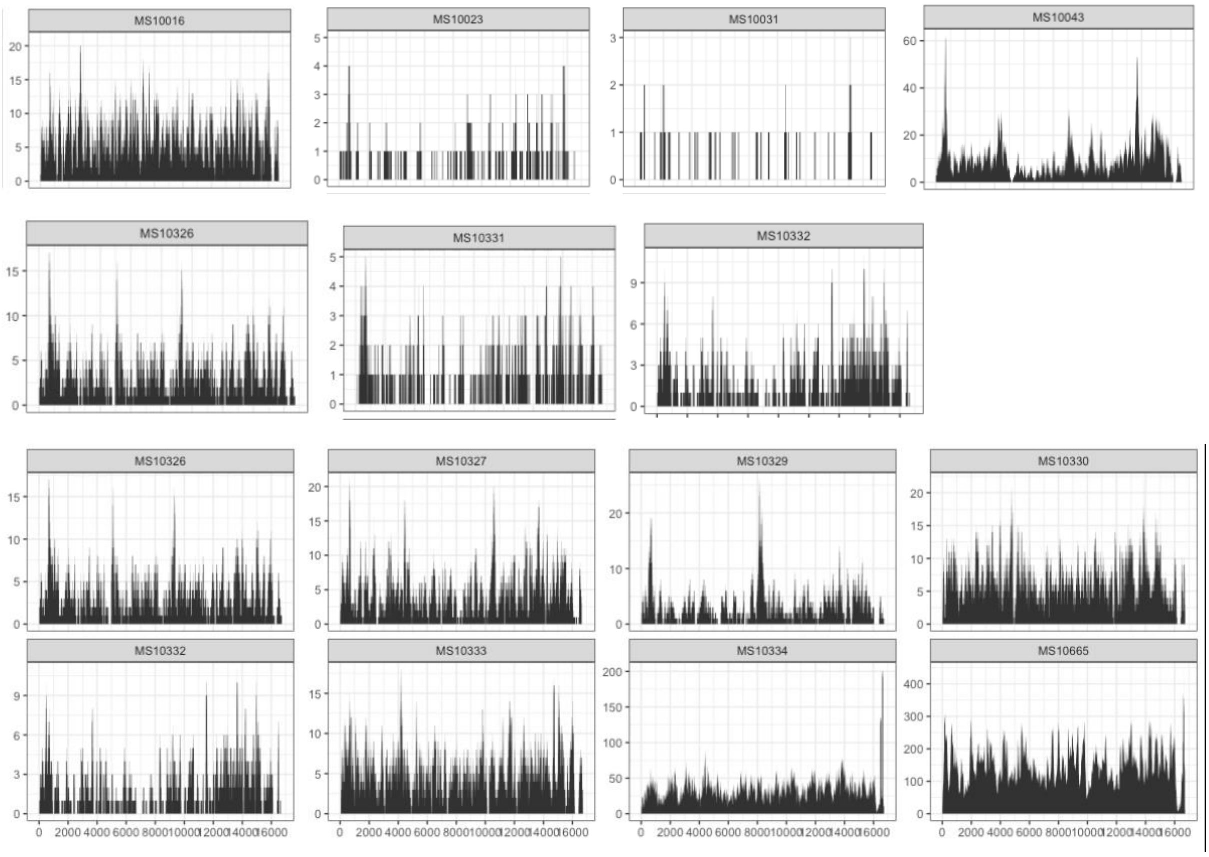

Figure S5: Read depth for the ancient dog sequences. The x-axis represents the length of the dog mitochondrial genome in base pairs. The y-axis is the number of reads covering each site of the mitogenome.

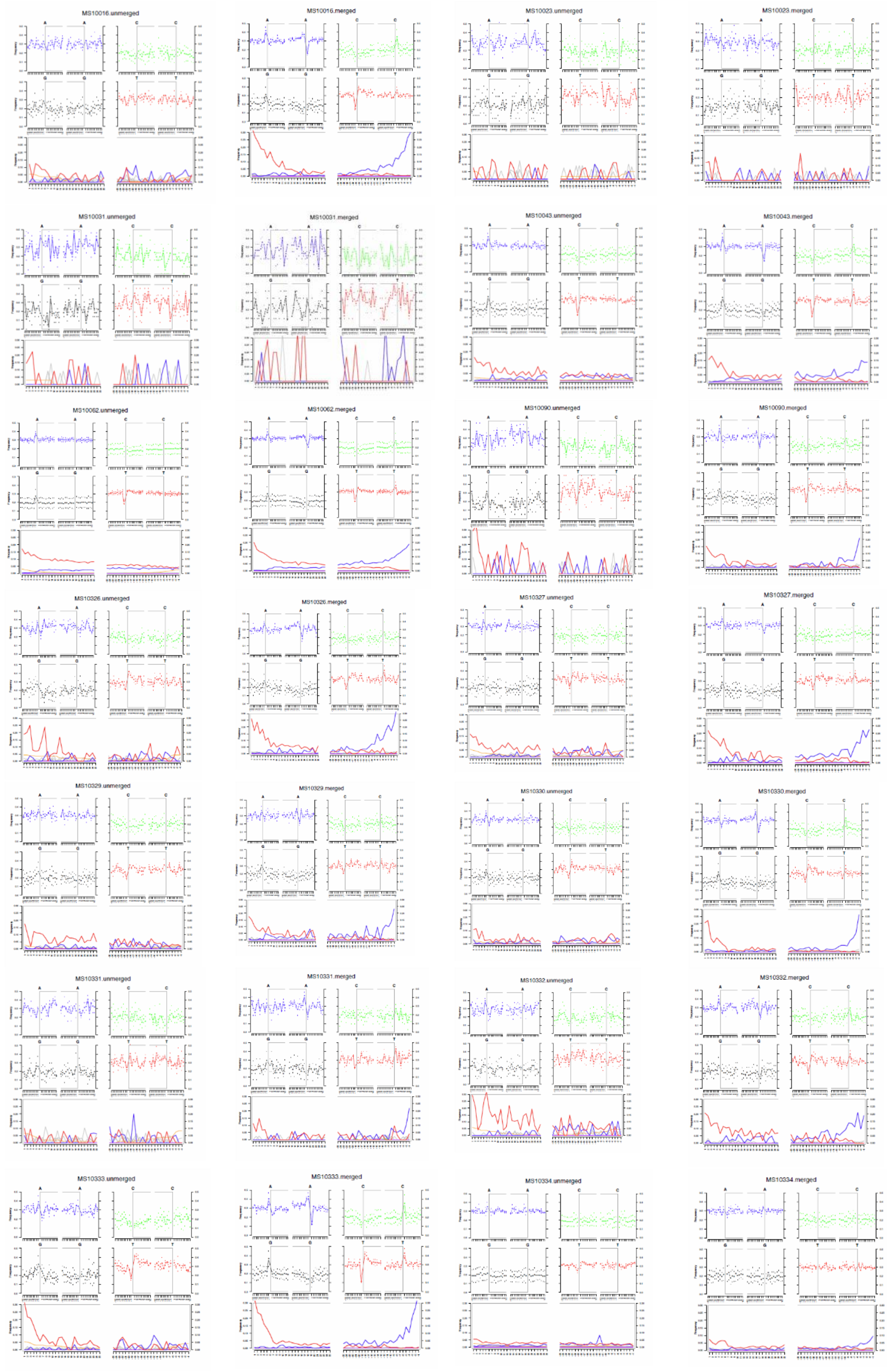
MS10016.unmerged
MS10016.merged
MS10023.unmerged
MS10023.merged
MS10031.unmerged
MS10031.merged
MS10043.unmerged
MS10043.merged
MS10062.unmerged
MS10062.merged
MS10090.unmerged
MS10090.merged
MS10326.unmerged
MS10326.merged
MS10327.unmerged
MS10327.merged
MS10329.unmerged
MS10329.merged
MS10330.unmerged
MS10330.merged
MS10331.unmerged
MS10331.merged
MS10332.unmerged
MS10332.merged
MS10333.unmerged
MS10333.merged
MS10334.unmerged
MS10334.merged

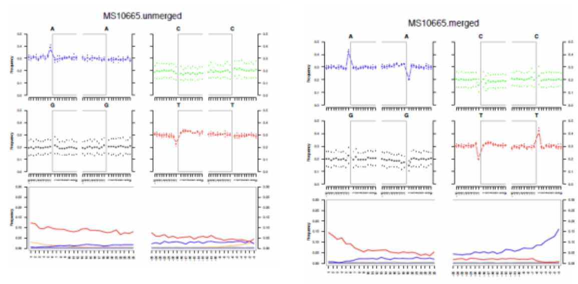

Figure S6. Ancient dog damage patterns. Top plots: Base frequency 50 and 30 of strand breaks. The gray brackets indicate start and end of molecules (strand breaks). Purines (A and G) show an elevated frequency before strand breaks. Bottom plots: C to T and G to A nucleotide misincorporations at the first and last 25 bases of endogeneous mtDNA fragments from the ancient dog sample [4].

References

1 Kumar, S., Stecher, G. & Tamura, K. MEGA7: Molecular Evolutionary Genetics Analysis version 7.0 for bigger datasets. *Molecular Biology and Evolution* **33**, 1870-1874 (2016).

2 Savolainen, P., Leitner, T., Wilton, A. N., Matisoo-Smith, E. & Lundeberg, J. A detailed picture of the origin of the Australian dingo, obtained from the study of mitochondrial DNA. *Proceedings of the National Academy of Sciences of the United States of America* **101**, 12387-12390, doi:10.1073/pnas.0401814101 (2004).

3 Leigh, J. W. & Bryant, D. popart: full-feature software for haplotype network construction. *Methods in Ecology and Evolution* **6**, 1110-1116, doi:10.1111/2041-210X.12410 (2015).

4 Sawyer, S., Krause, J., Guschanski, K., Savolainen, V. & Paabo, S. Temporal patterns of nucleotide misincorporations and DNA fragmentation in ancient DNA. *PLos One* **7(3)**: e34131 DOI 10.1371/journal.pone.0034131 (2012).

Supplementary Text

Modern dingo DNA extraction and sequencing

Four dingo blood samples (1.5mL each) were collected by Dr. Lisa Argilla at the Wellington Zoo (DW, DY, DB, DK) in August 2015. These were stored in EDTA for delivery to the University of Otago immediately following collection. Blood samples were kept in the freezer (-25°C) following arrival.

All DNA extraction and sequencing library preparation was carried out in a University of Otago modern DNA laboratory. 1000μL of blood was processed for each sample. As the blood was stored in EDTA, cells were extracted from the solution using 1x SSC buffer to ensure the EDTA would not inhibit the PCR. DNA was extracted from the cells using the MagJET magnetic bead (ThermoFisher) protocol (as per manufacturer's instructions). Samples were digested with Proteinase K for 4 hours prior to extraction. DNA was precipitated with isopropanol, washed with ethanol-based wash buffers and eluted in Elution Buffer (provided in Qiagen MinElute PCR Purification Kit).

Four long-range primer pairs (designed by Dr. Ann Horsburgh) were used to amplify the complete mitochondrial genomes of the Wellington Zoo dingoes. Table 1 gives the primer pairs used for amplification of each sample along with their sequences and binding positions.

Table 1. Primers used for long-range amplification of blood and tissue samples, and their relative position within the mitochondrial genome.

Samples:	Primer Pairs:	Primer Sequence:	5'-3' Binding Position:	Expected Length (bp):
Wellington Zoo dingoes	LR1F	TGGTATCACTCACCTACGACC	15,947-15,967	3457
	LR1.5R	CCTAACAAAGCCCCTTTTCTC	2656-2676	
	LR1.5F	CAACCTCCCCCAGTACGAAAG	2544-2564	3036
	LR1R	CGGCACTAGTCAGTTTCCAAA	5581-5601	
	LR2F	TGTCATCGTAACCGCCCATGA	5514-5534	5162
	LR2R	GGAGAGCTACTAGGAGTGGGA	10,676-10,696	
	LR3F	TGAAGCAACACTGATTCCGAC	10,569-10,589	5439
	LR3R	GACGGGCATATTCCCTGAGAG	15,998-16,018	
		[illegible]		

Long-range PCR products were amplified using KAPA Biosystems LongRange HotStart PCR Protocol (as per manufacturer's instructions). 2.0μL of template DNA was used in a 30.0μL reaction, with 1.5μL of each of the corresponding primers. All PCR reactions were run in a Bio-Rad iCycler. PCR products were viewed by gel electrophoresis using 2.5μL of PCR product on a 1% agarose gel to ensure DNA was present in the amplified fragments.

PCR products that showed expected lengths of DNA in the gel were purified using Qiagen MinElute silica spin columns, as the number of samples was small. This was done following the manufacturer's instructions.

Quantification of purified PCR products was done using Qubit dsDNA HS (High Sensitivity) Assay Kit and Qubit 3.0 flurometer (Life Technologies). For each sample, 199µL of Qubit dsDNA HS Buffer (0.2-100ng) buffer and 1µL of HS dsDNA dye. As the dye is light sensitive solution was made immediately prior to use. Standards are supplied in the kit.

Blunt end repair, ligation of sequencing adaptor and barcoding were carried out following Kircher at al. 2012, with modifications for Illumina sequencing adaptors. Samples were sonicated using diagenode Bioruptor Pico Sonication System to produce fragments c. 500bp in length. Purification of tissue sample libraries was performed using AMPure XP magnetic beads.
